# Improved therapeutic potential of tapentadol employing cationic exchange resins as carriers in neuropathic pain: evidence from pharmacokinetic and pharmacodynamics study

**DOI:** 10.1038/s41598-018-21214-2

**Published:** 2018-02-12

**Authors:** Manu Sharma, Ranju Soni

**Affiliations:** Department of Pharmacy, Banasthali Vidyapith, Banasthali, Rajasthan 304022 India

## Abstract

Current investigation was endeavoured to overcome problem of poor palatability and bioavailability of centrally acting analgesic, tapentadol (TAP) by formulating controlled release drug-resin complexes (DRCs). The technology encompassed in preparation of DRCs involved chemisorption of TAP to weak cationic resins (KyronT-134 and Tulsion335) by batch method. Various formulation variables like drug-resin ratio, pH, resin activation and swelling time were optimized to achieve maximum drug loading in DRCs. FT-IR, DSC, pXRD, *in vitro* release study under bio-relevant condition of mouth and *in vivo* sensory taste evaluation established formation of taste masked DRC whereas dissolution study assured prolonged drug release behaviour of optimized DRC. Among DRCs, TAP-KyronT-134 complex exhibited higher drug loading (80.89 ± 4.56%), stability and prolonged release profile (10 h) without any detectable amount of drug release under salivary conditions. Pharmacokinetic studies in wistar rats revealed increased T_max_ (2.67-fold), MRT (1.94-fold), elimination half-life (2.79-fold) and relative oral bioavailability (2.62-fold) of TAP on oral administration of optimized formulation compared to TAP solution. Furthermore, pharmacodynamics study confessed higher potential of DRC in attenuating chronic injury induced tactile allodynia for prolonged duration. In conclusion, the method developed is easily scalable and holds potential for commercialization with an evidence of obtaining more efficacious neuropathic pain management therapy.

## Introduction

Neuropathic pain is one of major healthcare disorder^1^. Persistent burning or shooting neuropathic pain arises due to direct or indirect injuries to somatosensory system that impairs working efficiency and quality of social life of sufferers. However, its onset mechanism is ambiguous which generates a great havoc in its treatment^2^. Moreover, origin of nerve injury varies and so the treatment options. Various therapeutic options clinically prescribed include tricyclic antidepressants, calcium channel α2-δ ligands, opioids and topical lidocaine in neuropathic pain management^3,4^. Although, development of tolerance and risk of associated side effects in long-term therapy limits their benefits^5–7^. Randomized clinical trials have shown consistent efficacy of tailored combination regimens by multimodal analgesia in individuals. However, drug-drug interaction and development of tolerability sometime complicates treatment options^8–10^. Therefore, effective neuropathic pain management demands an analgesic with multimodal action and safety in long-term use, which can improve working proficiency of an individual along with precise balance of analgesia and tolerability.

Tapentadol (TAP) is one of recent drug belonging to the class of centrally acting synthetic opioid analgesics approved by FDA^11^. It exhibits analgesic activity due to combined effect of agonistic activity at µ- opioid receptor and noradrenaline reuptake inhibition^12^. Dual mechanism of action bestowed TAP to diminish wide variety of acute and chronic pains including neuropathic, arthritic and back pain. Its multimodal functioning too reduces the risk of any undesirable drug-drug interaction^13,14^. Thus, TAP is a drug of choice for patients suffering from chronic pain conditions like arthritis or neuralgia who need to take therapy for longer duration of time. TAP is rapidly absorbed from its conventional tablets available commercially. However, extremely bitter taste and repetitive dosing (3–4 times a day) due to short half-life (4 h) of TAP eventually leads to non-adherence to its therapy^15^. Extended release formulations of TAP like Palexia SR and NUCYNTA^®^ ER are available in market. Marketed formulations like Palexia SR and NUCYNTA^®^ ER has bestowed limited success due to difficulty in swallowing among geriatric as well as paediatric patients. Patients are restricted to swallow NUCYNTA^®^ ER/Palexia SR tablets as whole. Since chewing, crushing, dissolving or dose adjustment by dividing tablets for patients with hepatic or renal impairment results in dose dumping leading to overdose and toxicity^16,17^. This necessitates for development of novel formulation approaches for improving the bioavailability and swallowability of TAP.

Nowadays, ion exchange resins (IERs) are gaining attention as a carrier for development of controlled release drug delivery systems, taste masking and stability improvement. The unique characteristic features of IERs like non-toxicity, non-irritancy, excreted unchanged from body, cost effectiveness and user friendly establishes them as suitable carrier system in drug delivery^18,19^. They serve as molecular sieves for drugs. Moreover, selection of resin for intended use depends on resin characteristics like acid or base strength, charge intensity, particle size and magnitude of cross-linking of resin. Drug loading capacity of resin depends on ion exchange capacity of resin, drug (molecular weight and its ionic nature) as well as process of drug loading i.e. batch or column method^19,20^. Literature reports various pharmaceutical grades of Polacrilex exhibit the property of taste masking, controlling release and improving stability by forming drug resin complexes (DRCs). Kyron T-134 and Tulsion335 are preferred pharmaceutical grade weak acid cation exchange polacrilex resins exhibiting higher exchange capacity (~10 meq/g) compared to sulphonic acid resins (~4 meq/g) or amine resins^21^. Chemically Kyron T-134 is the potassium salt of a unifunctional low cross linked carboxylic cation exchange resin prepared from methacrylic acid and divinylbenzene while Tulsion335 is a highly purified crosslinked polyacrylic copolymer in hydrogen form. Being high molecular weight, both resins are insoluble in all solvents as well as not absorbed inside body and excreted unchanged. These resins are commonly utilized as taste masking and stabilizing agents for bitter cationic drugs by forming drug resin complexes which remain stable in mouth^22,23^. However, drug release from complex is facilitated on contacting gastric fluid by exchanging with counter ions present in gastric fluid^24^. Thus, the adsorption-desorption features of KyronT-134 and Tulsion335 can be utilized in development of DRCs of pharmaceutical importance. Further DRCs can be dispensed in a variety of dosage forms like powder, chewing gums, suspension, conventional tablets or capsules^25–28^. Extended release resinates can maintain steady plasma drug levels, reduce frequency of multiple dosing, allows dose adjustment according to patient need and enhance adherence of patients to therapy^18,20^.

Aforementioned paradigm provides an opportunity for development of extended release TAP resinate complex to improve bioavailability and palatability of TAP. So far, to best of our knowledge, no literature reports are available explicating formulation of controlled release TAP resinates. Therefore, objective of current investigation was to prepare, characterize and evaluate taste masked extended release TAP- resin complex by ion exchange process to enhance bioavailability and compliance to TAP. Furthermore, pharmacokinetic and pharmacodynamics behaviour of extended release TAP-resin complex was determined to judge the potential of optimized formulation in relieving neuropathic pain.

## Results and Discussion

### Characterization of resins

Table 1 represents various characteristic properties like particle size, porosity, swelling index, cation exchange capacity of Kyron T-134 and Tulsion335 as received from manufacturer. It was observed that both resins were insoluble in water but swelled significantly. Higher hydration and swelling features of Kyron T-134 indicated higher regeneration of quiescent and coiled binding sites of resin for interaction with counter ions^28^. Thus, higher swelling index of resin contributed to its higher cation exchange capacity (Table 1).

**Table 1:** Characterization of cation exchange resins.

Resin	Particle size (µm)	Bulk density (ρb) (g/ml)	True density (ρt) (g/ml)	Porosity (%)	Swelling index (%)	Cation exchange capacity (meq/g)
KyronT-134	65.37 ± 7.36	0.736 ± 0.05	1.181 ± 0.07	36.02 ± 4.56	71.01 ± 8.96	10.92 ± 0.98
Tulsion335	43.25 ± 8.45	0.730 ± 0.04	1.134 ± 0.05	34.61 ± 3.56	35.06 ± 7.15	9.03 ± 0.87

### Optimization of formulation variables for maximum drug loading

The selection of suitable ion exchange resin for development of pharmaceutical formulations depend upon nature and characteristics of drug and resin respectively along with prerequisite of formulation^21,29^. TAP being a basic drug requires a weak acid cation exchange resin to form DRC. Kyron T-134 and Tulsion335 are composed of crosslinked polymeric matrix with carboxylic functional groups which serves as binding site for amine group of TAP to mask its bitter taste. Therefore, Kyron T-134 and Tulsion335 were selected for present study being inexpensive, inert and safe weak acid cation exchange resin with high drug loading capacity among various pharmaceutical grade resins^22,23^.

TAP was loaded on ion exchange resins like KyronT-134 or Tulsion335 by batch process. It is the most preferred method on laboratory scale for preparing drug resin complex. Since, batch process facilitates uniform swelling of fine sized resins and provides higher surface area for the process of ion exchange^24,28^.

IERs like KyronT-134 and Tulsion335 showed lower percentage of drug binding compared to activated resins respectively. Among the acid, alkali and both acid –alkali treated resin, KyronT-134 (80.89 ± 4.56%) showed maximum drug loading after acid-alkali activation whereas Tulsion335 (75.33 ± 3.09%) on acid activation (Table 2). The percentage drug loading increased significantly on resin activation. This might be due to rejuvenation of resin due to removal or neutralization of impurities adsorbed over the surface of resin during scale up processing, handling or storage by combined treatment of acid-alkali/acid solution^24^. After activation, exchangeable ion is H^+^ which has relatively lower selectivity coefficient compared to K^+^ ion for resin. Thus, increases complexation affinity of IER to drug as ionized amine groups of drugs has higher selectivity coefficient for resins compared to H^+^ ion^30^. This also ensures requirement of resin activation for faster and higher loading of drug on resin surface by providing availability of more activated exchangeable groups^18,20^.

**Table 2:** Effect of different formulation variables on drug loading capacity of resin complexes and amount of drug released under bio-relevant conditions of mouth and HCl buffer pH 1.2 in 3 min.

Formulation code	Resin	Resin activation method	Drug-resin ratio	pH	Swelling time (h)	Drug loading (%)	Selectivity Coefficient (K_D_)	Drug released (%)	
								Simulated salivary fluid pH 7.4	HCl buffer pH 1.2
F1	KyronT-134	Untreated resin	1:2	10.0	8	59.44 ± 4.39	34.75 ± 3.96	4.34 ± 0.97	12.34 ± 2.34
F2		Acid	1:2	10.0	8	70.97 ± 3.33	57.97 ± 3.01	—	7.89 ± 1.45
F3		Alkali	1:2	10.0	8	73.36 ± 2.89	65.29 ± 3.24	—	8.09 ± 2.34
F4		Acid- Alkali	1:2	10.0	8	80.89 ± 4.56	100.37 ± 4.68	—	3.15 ± 0.68
F5	Tulsion−335	Untreated resin	1:2	10.0	8	51.13 ± 2.69	33.98 ± 2.16	2.94 ± 0.93	10.34 ± 1.23
F6		Acid	1:2	10.0	8	75.33 ± 3.09	99.18 ± 4.18	—	3.45 ± 0.98
F7		Alkali	1:2	10.0	8	66.62 ± 4.12	64.83 ± 4.47	—	7.67 ± 1.01
F8		Acid- Alkali	1:2	10.0	8	52.60 ± 3.34	36.04 ± 3.95	4.16 ± 0.45	13.24 ± 2.09
F9	KyronT-134	Acid-Alkali	1:1	10.0	8	56.54 ± 3.12	30.85 ± 2.96	3.78 ± 0.79	13.09 ± 2.14
F10			1:3	10.0	8	81.01 ± 3.41	101.15 ± 4.87	—	3.08 ± 0.78
F11	Tulsion-335	Acid	1:1	10.0	8	50.97 ± 2.41	33.77 ± 3.24	3.84 ± 0.74	9.89 ± 0.92
F12			1:3	10.0	8	74.76 ± 3.63	96.21 ± 4.27	—	4.56 ± 0.87
F13	KyronT-134	Acid-Alkali	1:2	4.0	8	47.50 ± 3.14	28.22 ± 3.26	2.86 ± 0.07	8.97 ± 1.12
F14			1:2	6.0	8	58.47 ± 5.17	36.87 ± 5.47	3.78 ± 0.09	10.78 ± 1.34
F15			1:2	8.0	8	70.89 ± 4.62	62.91 ± 4.98	—	5.45 ± 0.88
F16			1:2	12.0	8	73.19 ± 5.32	61.26 ± 5.23	3.12 ± 0.07	9.41 ± 0.93
F17	Tulsion-335	Acid	1:2	4.0	8	40.73 ± 2.41	20.83 ± 3.14	3.16 ± 0.08	11.23 ± 1.23
F18			1:2	6.0	8	52.81 ± 4.64	35.26 ± 5.02	2.87 ± 0.03	9.78 ± 0.95
F19			1:2	8.0	8	65.08 ± 4.64	60.11 ± 5.23	—	3.45 ± 0.67
F20			1:2	12.0	8	67.49 ± 5.61	70.23 ± 5.98	—	3.09 ± 0.87
F21	KyronT-134	Acid-Alkali	1:2	10.0	6	71.07 ± 2.98	58.25 ± 3.46	3.39 ± 0.09	12.34 ± 1.10
F22			1:2	10.0	10	80.92 ± 4.72	100.56 ± 5.29	—	2.23 ± 0.45
F23	Tulsion-335	Acid	1:2	10.0	6	58.66 ± 4.55	46.09 ± 5.43	3.21 ± 0.03	6.78 ± 0.98
F24			1:2	10.0	10	74.71 ± 3.51	95.95 ± 5.12	—	2.01 ± 0.67

Drug resin ratio had also influenced the drug loading capacity of resin. The significant increase in drug loading was observed on varying drug resin ratio from 1:1 to 1:2 indicating that quantity of resin appreciably affects drug loading capacity (Table 2). The availability and accessibility of greater amount of resin surface for binding might had attributed to high drug loading. However, further increase of drug resin ratio above 1:2 had not appreciably affected drug loading due to saturation of resin binding sites with drug present in system^31^. Therefore, drug resin ratio 1:2 considered for further studies.

Alteration of pH from 4.0 to 12.0 of drug solution had significantly affected the complexation of TAP to activated IERs. Complexation of TAP to resin surface amplified from its aqueous solution with increase of pH from 4.0 to 10.0. However, further pH increase reduced the complexation behaviour (Table 2). Since pH affects intensity of ionization of TAP and resins along with solubility of TAP. Therefore, maximum ionization of drug (pKa of TAP ranges 9.34–10.45) and resin (pKa ranges 6.0–7.5) at pH 10.0 might have attributed maximum drug loading. However, further increase in pH (pH 12.0) had reduced drug loading due to reduced selectivity coefficient of amine drug (TAP) towards resin^24,30^. While strong affinity of H^+^ ions for binding to the –COO^−^ group of resin compared to drug contributed to lower drug loading at lower pH^25^.

Resin swelling time had also affected its drug loading capacity. KyronT-134 swollen for 6, 8 and 10 h with uniform stirring at 100 rpm exhibited drug loading of 71.07 ± 2.98%, 80.89 ± 4.56% and 80.92 ± 4.72% respectively. Similarly, Tulsion335 showed significant difference in drug loading with variation in swelling time (Table 2). The maximum drug loading for KyronT-134 (80.89 ± 4.56%) and Tulsion335 (75.33 ± 3.09%) was observed when respective resin was allowed to swell for 8 h in deionized water (pH 10.0) respectively. The results illustrated that hydrating and swelling properties of resins affected their drug loading capacity by rejuvenating inactive latent and coiled binding sites of resin^28,31^. These observations also confirmed that TAP complexation to resin surface is virtually a consequence of stoichiometric exchange of counter ions and thus affected by contact/swelling time^32,33^.

The relative affinity of both resins for drug and counter ions under various experimental conditions was affected by degree of hydration, availability of binding sites and pH of surrounding media (Table 2)^24^. Higher selectivity coefficient of drug was observed under equilibrium conditions of pH (10.0), swelling time (8 h) and drug resin ratio (1:2) for both resins.

Thus, optimized variables for preparation of TAP-KyronT-134 complex was acid-alkali activation of resin, drug resin ratio 1:2, pH 10.0 and resin swelling time of 8 h whereas acid activation of Tulsion335 was required to prepare optimized TAP-Tulsion335 complex.

### Drug resin equilibrium binding study

The amount of drug binding increased with increase of initial drug concentration (see supplementary Fig. S1). The increased driving force of concentration gradient of TAP might have contributed higher drug binding^33^. Isotherm models viz. Langmuir, Freundlich and Dubinin-Radushkevich (D-R) were used to evaluate equilibrium binding affinities of resins i.e. KyronT-134 and Tulsion335 for TAP under equilibrium conditions. The value of correlation coefficient (R^2^) for both resins was higher for Dubinin-Radushkevich (D-R) model [KyronT-134 (R^2^ = 0.934) and Tulsion335 (R^2^ = 0.924)] compared to Freundlich [KyronT-134 (R^2^ = 0.908) and Tulsion335 (R^2^ = 0.907)] and Langmuir model [KyronT-134 (R^2^ = 0.865) and Tulsion335 (R^2^ = 0.814)] respectively. The best fit to D-R isotherm model explained reversible adsorption of TAP on resin surface and resin porosity accounts for multisite drug adsorption on heterogeneous surfaces^34^. Higher numerical values of theoretical isotherm saturation capacity (Q_s_) for Kyron T-134 complex (415.71 mg/g) compared to Tulsion335 (371.89 mg/g) suggests its higher porosity. The difference in resin backbone structure as well as lower cross-linked structure of KyronT-134 might contribute to difference in its behaviour compared to Tulsion335^22,23^. D-R isotherm constant (K_ad_) for KyronT-134 and Tulsion335 was −0.00204 mol^2^ kJ^−2^ and −0.0022 mol^2^ kJ^−2^ respectively which was utilized to determine mean free energy change occurring during complexation. The mean free energy change arising during movement of 1 mol of TAP ions in solution to resin surface from infinity, was calculated by following mathematical relationship

1 $${\rm{E}}={(-2{{\rm{K}}}_{{\rm{ad}}})}^{-1/2}$$

It was observed that mean free energy change occurring during TAP adsorption on resin surface was highest for KyronT-134 (15.66 kJ/mol) followed by Tulsion335 (15.07 kJ/mol). Numerical value of E (between 15–16 kJ/mol) provides evidences for chemisorption of TAP on resin surface because of exchange of ions^35^. Thus, D-R isotherm confirmed that drug loading during drug-resin complex formation was a function of penetration of counter ion across porous resin matrix along with chemisorption.

### Molecular characterization of DRC

**Figure 1:**
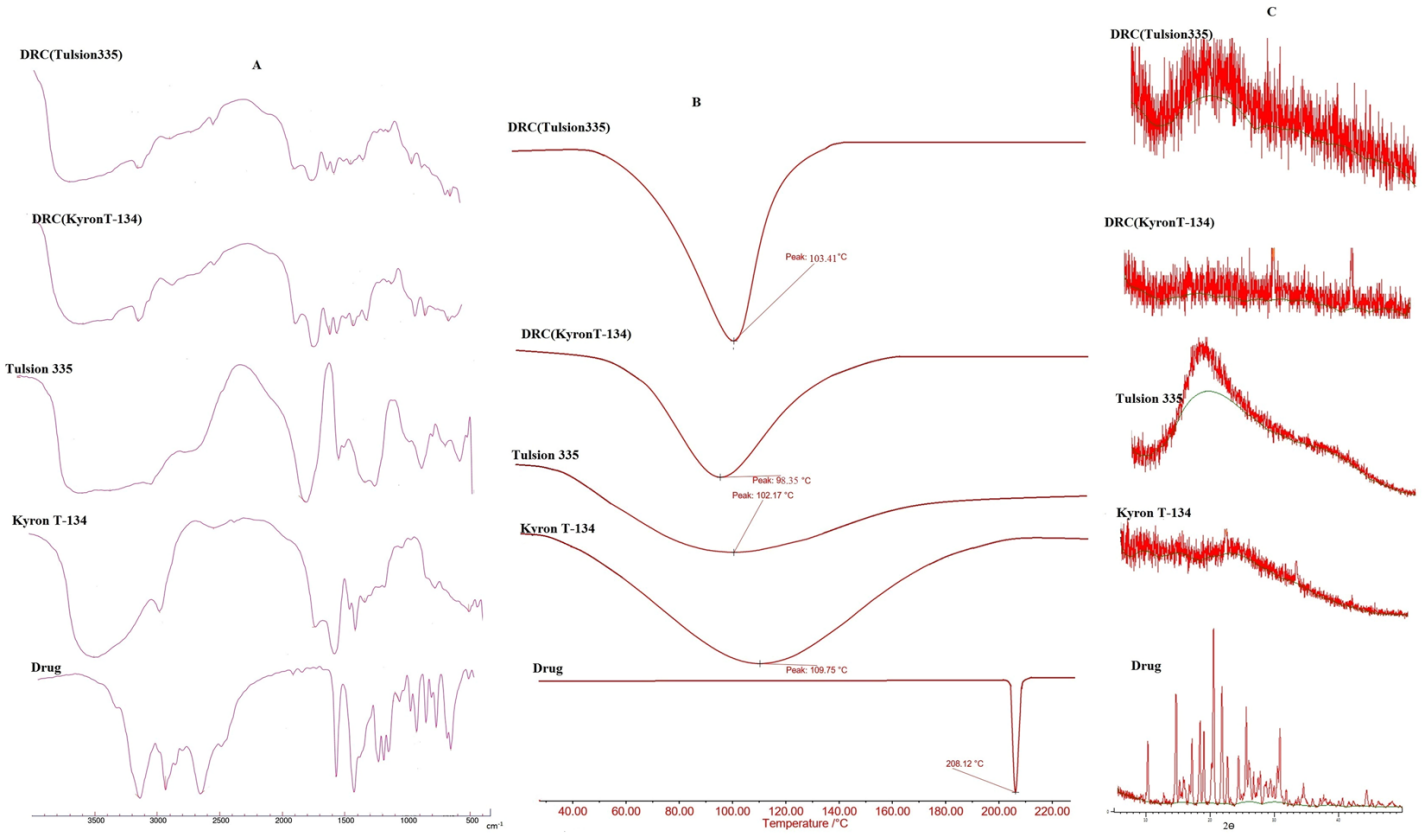
(**A**) FTIR spectra of drug, KyronT-134, Tulsion335, TAP-KyronT-134 complex and TAP-Tulsion335 complex respectively. (**B**) DSC thermograms of drug, KyronT-134, Tulsion-335 and optimized DRCs of KyronT-134 and Tulsion335 respectively. (**C**) Powder X- ray diffractograms of drug, KyronT-134, Tulsion335, TAP- KyronT-134 complex and TAP-Tulsion-335 complex respectively.

Figure 1A represents FT-IR spectra of TAP, KyronT-134, Tulsion335, drug KyronT-134 complex and drug Tulsion335 complex. The spectra of KyronT-134 showed a broad peak of O-H stretching vibration of carboxylic acid at 3464 cm^−1^, C-H stretching at 2947 cm^−1^, C=O stretching at 1761 cm^−1^ and C-O stretching at 1328 cm^−1^. Similarly, Tulsion335 also showed broad peak of O-H stretching at 3481 cm^−1^ and C=O stretching at 1711 cm^−1^. TAP spectrum had sharp peak for C-O stretching vibration at 1259 cm^−1^, CH_2_ bending at 1454 cm^−1^, C-N stretching at 1178 cm^−1^ and peaks at 878 cm^−1^ and 797 cm^−1^ corresponds to =C-H out of plane bending of substituted benzene. The peak corresponding to C-N stretching at 1250–1020 cm^−1^ and C-O stretching at 1320–1210 cm^−1^ in DRC indicated the presence of drug in complex. TAP hydrochloride as the salt of tertiary amine showed the prominent peak at 2682 cm^−1^ for N-H stretching vibration in its spectra. However, spectra of DRCs were devoid of N-H stretching vibration confirming the formation of complex between drug and resin^36^.

Thermal behaviour of TAP, resins and DRCs was evaluated by differential scanning calorimetry. DSC thermogram of TAP displayed a sharp endothermic peak at 208.12 °C indicating its crystalline behaviour. Resins like KyronT-134 and Tulsion335 showed broad peak at 109.75 °C and 102.9 °C respectively (Fig. 1B) in their thermogram. However, DRC thermograms showed complete disappearance of endothermic peak of TAP indicating reduced crystallinity of drug because of complexation with ion exchange resin.

The crystallinity of TAP, KyronT-134, Tulsion335, TAP-KyronT-134 resin complex and TAP-Tulsion335 was accessed by X-ray diffractometer (Fig. 1C). X-ray diffractogram of TAP showed intense and sharp peaks confirming crystalline behaviour of drug while presence of diffused peaks assured amorphous nature of resins. Diffractograms of DRCs had also showed diffused peaks that were devoid of crystalline peaks of TAP. These finding illustrated monomolecular dispersion and complete embedding of drug in resin matrix. Thus, characterization techniques i.e. FT-IR, pXRD and DSC studies established the formation of drug resin complex along with its amorphous nature.

### Swelling studies

The optimized drug resin complexes prepared with KyronT-134 and Tulsion335 had swelled to 50.12 ± 2.14% and 36.12 ± 4.28% respectively after 2 h, 53.26 ± 4.12% and 38.06 ± 5.81% of their original weight in HCl buffer pH 1.2 after 4 h while 63.58 ± 3.67% and 40.21 ± 3.87% respectively after 8 h. This confirms hydration and swelling of DRC in dissolution media. Particle size analysis of optimized batches of DRC i.e. TAP-KyronT-134 and TAP-Tulsion335 showed increase in their particle size from 165.64 ± 11.23 µm and 74.32 ± 9.12 µm to 2.99 ± 0.11 mm and 2.13 ± 0.18 mm respectively when incubated at 37 °C for 2 h in HCl buffer pH 1.2. Furthermore TAP-KyronT-134 and TAP-Tulsion335 DRC immersed in HCl buffer showed reduction of surface charge from −11.23 ± 1.56 mV and −8.97 ± 1.19 mV to −1.235 ± 0.01 mV and −1.261 ± 0.01 mV respectively. Zeta potential near to zero indicates instability of dispersion due to aggregation of particles in slurry^37^. Thus, swelling of particles along with their aggregation contributed to increase in particle size of respective DRC in acidic buffer. Optical microscopy confirmed swelling as well as aggregation of DRC in HCl buffer pH 1.2 (see Supplementary Fig. S2). This might be due to protonation of carboxylic group of resin at pH below its pKa ~6.0 facilitating increase in Van der Waals force of attraction and reduction in electrostatic repulsive forces. Literature reports that particles of size greater than 0.5 mm remain in stomach until fragmented to smaller size^38^. Thus, DRCs might show gastro-retentive behaviour due to their swelling behaviour along with aggregation of particles of DRCs to a size greater than 0.5 mm. However, aggregated particles of DRCs in HCl buffer pH 1.2 showed de-aggregation as pH of dispersion raised to 6.0 due to rehabilitation of negative charge of carboxylic groups of resin because of their deprotonation^39^.

### Dissolution studies

During *in vitro* release study under bio-relevant conditions of mouth, optimized DRCs had not released any detectable amount of drug kept for 3 min in simulated salivary fluid pH 7.4 (Table 2). Higher binding affinity between ionized resin and drug at pH 7.4 as well as short interval of exposure time with small cation concentration (≈40 mEq/l) in simulated salivary fluid cannot alter the ion exchange process^31^. Thus, TAP complexation with KyronT-134 or Tulsion335 can mask bitter taste of TAP by preventing drug release.

**Figure 2:**
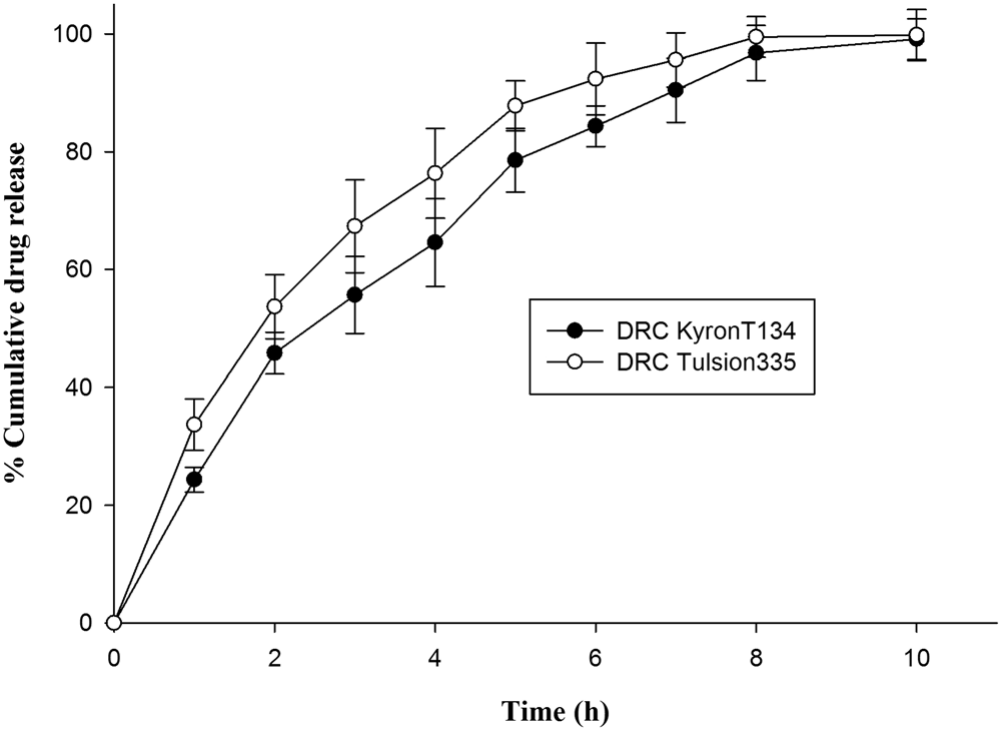
Dissolution profile of optimized DRCs of KyronT-134 and Tulsion335 respectively.

On behalf of swelling and aggregating property of DRCs in HCl buffer pH 1.2, formulation might show gastro-retentive behaviour^38,39^. Therefore, release behaviour of DRCs was evaluated in HCl buffer pH 1.2. TAP-KyronT-134 and TAP-Tulsion335 complex facilitated almost 46% and 54% drug release respectively within 2 h while complete drug release in 10 h during dissolution study (Fig. 2). Comparative evaluation of results of *in vitro* drug release study under bio-relevant conditions of mouth and HCl buffer pH 1.2 demonstrates that TAP displacement from DRC is highly dependent on pH of surrounding media similar to drug loading in resin (Table 2). Drug release in stomach follows equation

2 $${{\rm{Resin}}}^{-}{{\rm{Drug}}}^{+}+{{\rm{H}}}^{+}\to {{\rm{Resin}}}^{-}{{\rm{H}}}^{+}+{{\rm{Drug}}}^{+}$$

pKa value of resins has significant influence on drug release rate from drug-resinate in gastric fluid as ionization of resins is strongly influenced by solution pH^24^. The weak cation exchange resins exist in non-ionized state as the free acid at pH <pKa (~6.0). Thus, relative higher affinity of resins for H^+^ ion in acidic aqueous environment facilitated initial faster desorption of drug from DRC surface (Fig. 2)^29^. Both TAP-KyronT-134 and TAP-Tulsion335 complex showed hydration, swelling and aggregation in dissolution fluid as contact time increased (see Supplementary Fig. S2). Thus, swelling time of complex and slower diffusion of exchange ions (H^+^/drug ion) across hydrated resin matrix due to increased path length gradually slowed down the release rate and prolonged the duration of drug release^32,40,41^.

TAP release data presented in Fig. 2 from optimized TAP-KyronT-134 complex and TAP-Tulsion335 complex was analysed to determine mechanism of drug release by different release kinetics models like zero order, first order, Higuchi and Korsmeyer-peppas. Drug release data showed best fit to Korsmeyer-peppas equation as confirmed by comparing regression coefficient of various release kinetic models. The regression coefficient values for different release kinetic models i.e. zero order are R_0_^2^ = 0.896 and 0.814 for TAP-KyronT-134 complex and TAP-Tulsion335 complex, first order: R_1_^2^ = 0.832 and 0.795, Higuchi: R_H_^2^ = 0.937 and 0.976 and Korsmeyer-peppas equation: R_K_^2^ = 0.975 and 0.997 respectively. Numerical value of diffusion exponent (n) for Korsmeyer-peppas equation of optimized drug-KyronT-134 and TAP-Tulsion335 complex was 0.705 and 0.635 respectively. The ‘n’ value between 0.5 < n < 1 suggest anomalous behaviour of drug release referring to combination of diffusion and dissolution phenomenon. Thus, *in vitro* release studies revealed that desorption of drug from DRC and diffusion of dissolved drug across the swelled matrix of DRC controlled the drug release^24^.

### Stability study of DRC

Stability data of TAP-KyronT-134 complex and TAP-Tulsion335 complex under real time and accelerated stability conditions is shown in Table 3. Stability indicators of DRCs were *in vitro* release study under bio-relevant conditions of mouth and gastric environment during study. The optimized DRCs of KyronT-134 and Tulsion335 showed almost 99% drug release in 10 h in HCl buffer pH 1.2. However, no significant amount of drug was released from DRC in simulated salivary fluid pH 7.4 even after storage at 30 ± 2 °C/60 ± 5% RH for 3 months. On the other hand, drug-Tulsion335 complex stored under accelerated conditions for 6 weeks facilitated significant amount of drug release in simulated salivary conditions and 98% drug release in 1 h in simulated gastric conditions. DSC studies further confirmed the breaking of drug resin complex as DSC thermogram showed endothermic peak at 201.81 °C for drug. However, colour of all the DRCs remained unchanged when observed visually. Thus, results of stability study evidenced higher stability of optimized TAP-KyronT-134 complex.

**Table 3:** Stability studies of optimized DRCs under room temperature (30 ± 2 °C/60 ± 5% RH) and accelerated (40 ± 2 °C/75 ± 5% RH) storage conditions.

S. No.	Storage condition	Drug resin complex	Storage time (month)	*In vitro* drug release (%)		
				HCl buffer pH 1.2		Simulated salivary fluid pH 7.4 (3 min)
				**2 h**	**8 h**	
1	Room temperature (30 ± 2 °C/60 ± 5% RH)	Drug-KyronT-134 complex	1	42.69 ± 3.54	86.23 ± 4.65	No release
			1.5	43.36 ± 2.69	88.01 ± 5.46	
			3	46.24 ± 5.89	90.45 ± 4.78	
		Drug-Tulsion 335 complex	1	54.45 ± 2.34	99.24 ± 5.64	No Release
			1.5	55.56 ± 5.64	97.65 ± 4.52	
			3	56.21 ± 3.21	99.19 ± 6.58	
2	Accelerated temperature (40 ± 2 °C/75 ± 5% RH)	Drug-KyronT-314 complex	1	43.25 ± 2.39	85.31 ± 5.12	No release
			1.5	45.58 ± 4.15	89.65 ± 7.25	
			3	46.32 ± 5.46	87.69 ± 6.54	
		Drug-Tulsion 335 complex	1	78.04 ± 6.52	99.19 ± 3.89	No Release
			1.5	98.04 ± 3.45	99.36 ± 5.47	5.89 ± 2.45
			3	99.18 ± 4.21	99.68 ± 8.19	

### *In-vivo* taste evaluation

During *in vivo* taste evaluation study, TAP solution having different drug concentration i.e. 10 µg/ml, 20 µg/ml, 30 µg/ml and 40 µg/ml had shown concentration dependent reduction of licking behaviour i.e. 24.57 ± 9.01%, 48.24 ± 10.61%, 68.51 ± 9.07% and 83.64 ± 7.19% respectively in animal model compared to distilled water. However, TAP-KyronT-134 complex had not elicited reduction in licking behaviour with respect to distilled water. Thus, *in vivo* sensory taste evaluation study confirmed that preparation of TAP-KyronT-134 complex could conceal TAP’s unpalatable taste. The results obtained are in correlation to *in vitro* release studies of optimized DRC under bio-relevant conditions of mouth.

### Pharmacokinetic profile

**Figure 3:**
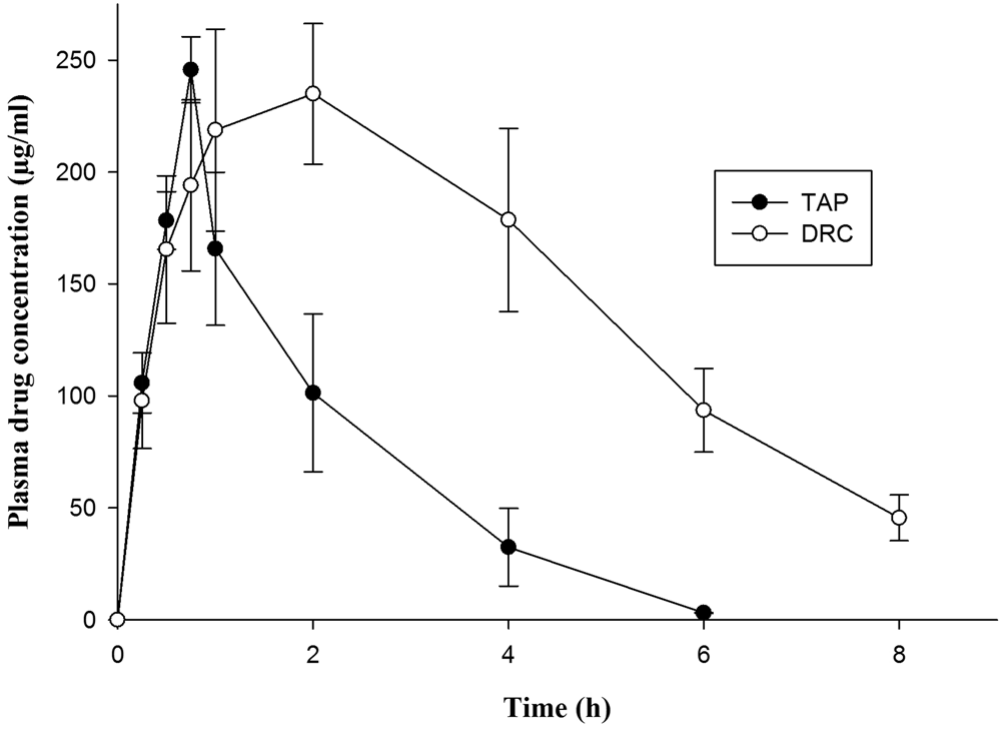
Plasma drug concentration profile of TAP after oral administration of TAP solution and optimized DRC in wistar rats respectively.

Figure 3 and Table 4 represents plasma concentration - time profile and pharmacokinetic parameters viz. C_max_, T_max_, t_1/2,_ AUC_0–24 h_, MRT of TAP and TAP-KyronT-134 complex following oral administration of single dose equivalent to 3 mg/kg drug respectively. Peak plasma concentration of pure drug and DRC had no significant difference. However, T_max_ achieved by DRC was 2.67-fold higher compared to pure drug indicating sustained absorption of drug from formulation under *in vivo* condition^37,41^. Plasma concentration – time profile of DRC showed significantly higher plasma drug concentration after 2 h of its post administration compared to free drug. The extended absorption of controlled release TAP from DRC might have contributed higher plasma drug concentration with simultaneous elimination of already absorbed drug. Since only fraction of drug absorbed inside body is subjected to elimination. TAP exhibited rapid systemic clearance as indicated by its elimination half-life (t_1/2_) (0.89 ± 0.06 h) owing to its poor plasma protein binding^42^. However, t_1/2_ of TAP extended to 2.79 times on oral administration of DRC demonstrates slower systemic clearance, which was further confirmed by higher MRT (1.94 times) of DRC compared to free drug. Thus, slower release leading to continuous extended TAP’s absorption from DRC had slowed down elimination and prolonged MRT^43^. The mean observed plasma (AUC)_0–24 h_ of DRC was also significantly improved to 2.62-fold compared to pure drug indicating improved bioavailability of TAP from DRC. Thus, results of pharmacokinetic study also serve as an evidence to validate that TAP complexed with resin worked as sustained release drug delivery system and can improve the performance of TAP in pain management.

**Table 4:** Pharmacokinetic parameters obtained after oral administration of optimized DRC (TAP-KyronT-134 complex) and TAP solution at a dose equivalent to 3 mg/Kg TAP.

Parameters	TAP solution	Optimized DRC
T_max_ (h)**	0.75 ± 0.19	2.00 ± 0.36
C_max_ (µg/ml)	245.67 ± 68.99	234.98 ± 79.16
t_1/2_ (h)**	0.89 ± 0.06	2.49 ± 0.08
k_e_ (h^−1^)**	0.78 ± 0.03	0.28 ± 0.01
AUC_(0–24)_ (µg h/ml)**	456.09 ± 72.01	1193.58 ± 161.76
AUMC (µg h^2^/ml)**	846.05 ± 84.13	4294.27 ± 164.41
MRT (h)*	1.86 ± 0.24	3.60 ± 0.38
Relative bioavailability (%)	—	261.69

^*^p < 0.05 level of significant difference; **p < 0.001 level of significant difference.

### Behavioural mechanical anti-allodynia activity

**Figure 4:**
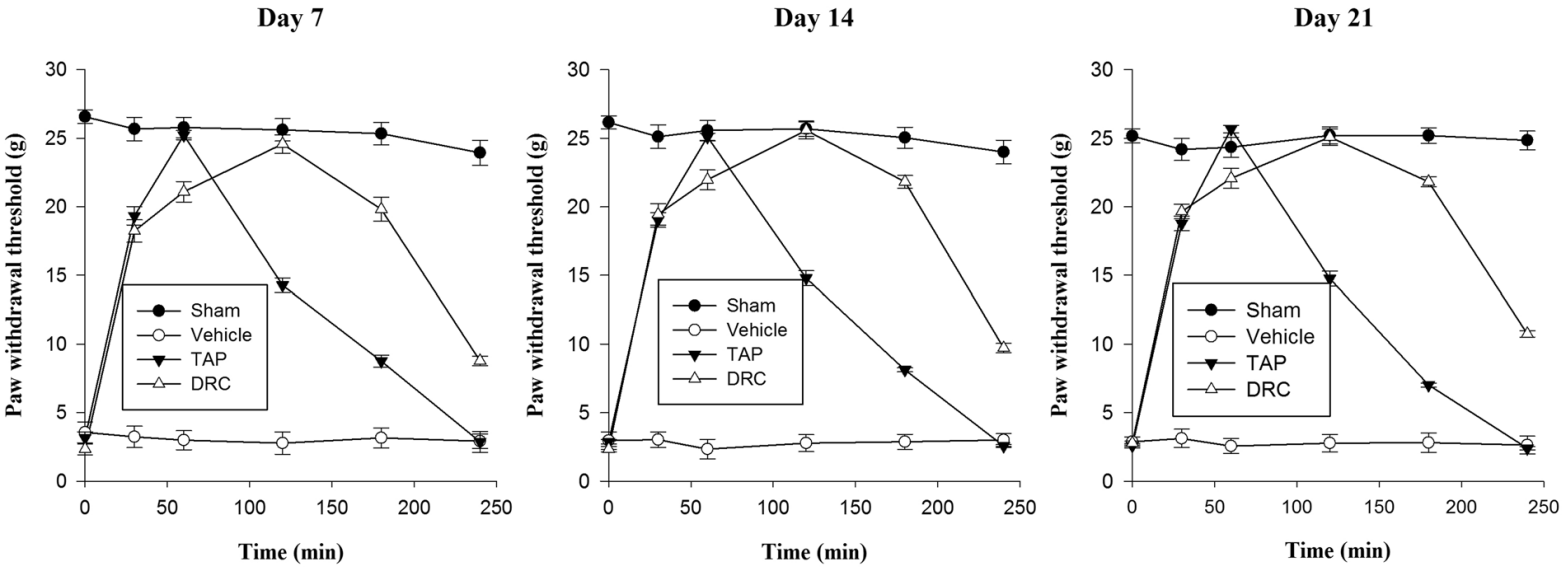
Impedance of mechanical allodynia on oral administration of TAP solution and optimized DRC (TAP-KyronT-134 complex) in wistar rats with chronic constriction injury induced neuropathic pain during study period.

Wistar rats with chronic constriction injury developed marked hypersensitivity on lateral surface of hind paw with innocuous mechanical von Frey probe. Hypersensitivity to mechanical stimulus persisted throughout the experimental period^3,44^. This might be contributed by activation of resident macrophages, schwann cells and cells adjacent to nerve lesion to secrete pro-inflammatory mediators initiating inflammatory reactions^45^. Paw withdrawal threshold of sham control varied from 23.87 ± 0.79 g to 26.57 ± 0.45 g during study period of 21 days. Oral administration of TAP solution or DRC dispersion had significantly attenuated chronic injury induced tactile allodynia. However, vehicle had not modulated surgery induced mechanical allodynia (Fig. 4). This reduction in pain probably is the synergistic activity of TAP on µ-opioid receptor and noradrenaline reuptake inhibition (NRI). Earlier published reports have also confessed TAP’s analgesic effect in chronic constriction injury of sciatic nerve due to its predominant effect on noradrenaline reuptake inhibition along with agonism of µ-opioid receptor^46^.

The intensity and time span of anti-hypersensitivity effect of oral administration of TAP or DRC remained almost same during the test period. This indicates that daily oral dosing of TAP solution or DRC dispersion had not induced tolerance or enhanced anti-hypersensitivity (Fig. 4). DRC dispersion had significantly prolonged the duration of anti-allodynia response to von Frey probe that shows consistency with pharmacokinetic profile of DRC. Thus, TAP-resin complex formulation offers a useful alternative to overcome the repetitive dosing of TAP, a short acting analgesic effective in neuropathic pain.

## Methods

### Materials

TAP was obtained as a gift sample from MSN Pharma Pvt Ltd, Hyderabad, India. Corel Pharma Chem and Thermax India Ltd, India provided KyronT-134 and Tulsion335 as gift sample respectively. Potassium di-hydrogen phosphate, disodium hydrogen orthophosphate, dipotassium hydrogen orthophosphate, formic acid, carboxymethyl cellulose and hydrochloric acid were purchased from CDH, India. Analytical grade chemicals and reagents were used throughout study. Double distilled water was used during study.

### Characterization of resins

Kyron T-134 and Tulsion335 was characterized for particle size (Malvern Metasizer, UK), bulk density (Veego Densitometer, India), true density (Smart Pycno 30, Smart Instruments, Mumbai) and swelling index. Both resins were dispersed in liquid paraffin respectively before estimating particle size. For measuring swelling index of resins, resins (1.0 g) were soaked in fixed quantity of water (25 ml) for 24 h respectively. The percentage swellability of resins was determined using following equation^47^

3 $$ \% {\rm{Swelling}}\,{\rm{index}}=[({\rm{Vf}}-{\rm{Vi}})/{\rm{Vi}}]\times 100$$

where Vf and Vi is the final volume of resin after swelling and initial volume of resin respectively.

The effective percentage porosity of resins after determining bulk density and true density was determined using formulae^48^

4 $$ \% {\rm{\varepsilon }}=[({{\rm{\rho }}}_{{\rm{t}}}-{{\rm{\rho }}}_{{\rm{b}}})/{{\rm{\rho }}}_{{\rm{b}}}]\times 100$$

where ε = effective porosity, ρt = true density and ρb = bulk density.

### Cation exchange capacity determination

An accurately weighed amount of resin (1.0 g) was dispersed in 1 N HCl (50 ml) with uniform stirring for 10 min. Samples were filtered and washed with excess distill water to drain acid. Acid treated resins were immersed in 0.1 N NaOH solution containing 5% sodium chloride. Samples were kept overnight to convert H^+^ sites of resin into Na^+^. Supernatant was withdrawn and residual amount of sodium hydroxide solution was back titrated with 0.1 N HCl using phenolphthalein as indicator^49^.

5 $${\rm{Cation}}\,{\rm{exchange}}\,{\rm{capacity}}\,({\rm{meq}}/{\rm{g}})=({{\rm{N}}}_{{\rm{NaOH}}}\,{{\rm{V}}}_{{\rm{NaOH}}}-{{\rm{N}}}_{{\rm{HCl}}}\,{{\rm{V}}}_{{\rm{HCl}}})/{\rm{w}}$$

where N_NaOH_, V_NaOH,_ N_HCl_, V_HCl_ is the normality of standardized NaOH, initial volume of NaOH solution added, normality of standardized HCl solution and volume of HCl consumed during back titration respectively. W is the weight of dried resin.

### Resin activation

Resins were purified before DRC preparation. Briefly, an accurately weighed amount of resins (7 g) was dispersed in 1 N HCl (100 ml) and stirred for 10 min at 300 rpm respectively. Resin dispersion was vacuum filtered. The collected residue was rinsed off with distilled water until attained neutral pH and dried overnight at 50 °C. Dried resins were sieved (sieve number 60) before further use. Similarly, resins were treated with alkali and acid-alkali to investigate the suitable method of activation. Briefly, alkali activation of resins was done similarly using 1 N NaOH instead of 1 N HCl. Resins were also activated with subsequent treatments of acid (1 N HCl) and alkali (1 N NaOH) in acid-alkali activation method^28^.

### Preparation of drug-resin complex (DRC)

Activated cationic exchange resins viz. KyronT-134 and Tulsion335 were used to prepare TAP- resin complex by batch method^28^. Briefly, activated resin was dispersed in distilled water (200 ml) adjusted to pH 10.0 with subsequent addition of TAP (3.5 g). The resulted dispersion was uniformly stirred (100 rpm) at 25 °C for 8 h to facilitate complete swelling and complexation of resin with drug. On filtration, collected DRC was washed with excess amount of distilled water followed by drying at 40 °C in a desiccator. Filtrate was analysed for drug content by U.V/Visible spectrophotometer (UV/Vis 3000^+^, LabIndia) at 271 nm. Different batches of DRC prepared to optimize resin activation method, drug-resin ratio, pH and contact time for swelling of resin are coded in Table 2. The percentage drug loading was estimated using following formula

6 $${\rm{Drug}}\,\text{Loading}( \% )=[({{\rm{T}}}_{{\rm{D}}}-{{\rm{T}}}_{{\rm{F}}})/{{\rm{T}}}_{{\rm{D}}}]\times 100$$

where T_D_ is the total amount of drug added and T_F_ is the total amount of free drug.

### Measurement of selectivity coefficient

The relative affinity of resin to a particular ion under various parameters of formulation optimization was measured to determine the equilibrium condition of formation of DRC. A series of 24 dispersions of resin corresponding to batches of formulation (F1–F24) were prepared in 0.154 M sodium chloride solution (200 ml) adjusted to respective pH. Resin dispersions were stirred (100 rpm) at 25 °C for respective duration of swelling time. Subsequently, resin was separated by filtration. Resin obtained was re-dispersed in 0.1 N HCl (200 ml) for 2 h to elute cations bound in resin phase and filtered. Aliquots (20 ml) withdrawn from equilibrium and acid elution filtrate solution were evaporated to constant weight. The weight concentration in equilibrium (C_s_) and elution solution (C_r_) were converted in equivalents per litre by dividing respective weight obtained with average molecular weight of solids^50^. The selectivity coefficient was calculated using equation^24^

7 $${\rm{Selectivity}}\,{\rm{Coefficient}}({{\rm{K}}}_{{\rm{D}}})=({{\rm{T}}}_{{\rm{r}}}\times {{\rm{C}}}_{{\rm{s}}})/({{\rm{T}}}_{{\rm{s}}}\times {{\rm{C}}}_{{\rm{r}}})$$

where T_r_, T_s_, C_r_ and C_s_ are total bound drug concentration in resin, total free drug concentration in solution, counter ion concentration in resin and counter ion concentration in solution respectively. Concentration of drug and counter ions were expressed in meq/l. T_r_ and T_s_ drug concentration determined for calculation of drug loading was used for estimation of selectivity coefficient.

### Drug- resin equilibrium binding study

The equilibrium drug resin binding study was performed by batch equilibrium procedure^33^. Activated resin (0.3 g) was weighed precisely and dispersed in TAP solution of varying concentration (50 mg/l–500 mg/l) under equilibrium conditions of pH (10.0) and contact time (8 h) for swelling of resin at 25 °C with uniform stirring (100 rpm). DRCs collected as residue on filtration were washed with excess amount of water to remove free drug followed by drying in desiccator at 40 °C. Filtrates collected were diluted with distilled water and analysed spectrophotometrically to estimate unbound drug content at 271 nm. The amount of drug bound to resin, Q_e_ (mg/g) was determined using equation^33,34^

8 $${{\rm{Q}}}_{{\rm{e}}}=({{\rm{C}}}_{{\rm{o}}}-{{\rm{C}}}_{{\rm{e}}}){\rm{V}}/{\rm{w}}$$

where Co and Ce are the initial and equilibrium drug concentration (mg/l) of solution respectively, V is drug solution volume (l) and w is resin weight (g).

Distribution of drug molecules between their solution (liquid phase) and resin (solid phase) during equilibrium drug resin binding process can be predicted by adsorption isotherm models viz. Langmuir, Freundlich and Dubinin-Radushkevich^34^. The regression coefficient (R^2^) was calculated to determine the best-fit model for predicting the mechanism of drug binding.

## Characterization of DRC

### Fourier transform infrared (FT-IR) spectroscopy

FTIR spectra were recorded for TAP HCl, KyronT-134, Tulsion335 and optimized DRCs (F4 and F6) in spectral region of 4000–400 cm^−1^. KBr pellets of samples developed, crimped and scanned using FTIR spectrophotometer (Shimadzu FTIR 8300 spectrophotometer).

### Differential scanning calorimetry (DSC)

Thermal behaviour of drug, KyronT-134, Tulsion335 and optimized DRCs (F4 and F6) was determined using differential scanning calorimetry (DSC-204 F1 phoenix) to access the effect of complexation of drug to resin on thermal behaviour of TAP. Samples hermetically sealed in aluminium pans were heated over a scanning temperature range from 30–250 °C at a rate of 10 °C/min under dynamic nitrogen purging to prevent overheating of instrument.

### Powder X-ray diffraction (pXRD)

pXRD spectra of TAP HCl, KyronT-134, Tulsion335 and optimized drug resin complexes were obtained using X-ray diffractometer (XPERT-PRO, PAN analytical, Netherlands) using CuKα radiation emitted from Cu line as a radiation emitting source at a voltage of 45 KV and 25 mA current. Diffractograms of samples were recorded over 2θ range from 5° to 50° at 25 ± 1 °C.

### Swelling index determination

The extent of swelling of optimized F4 and F6 DRCs was determined by suspending DRCs (50 mg) in HCl buffer pH 1.2 (10 ml) respectively. The percentage swelling of DRCs was determined by measuring weight of swelled DRCs after filtration and bloating off excess buffer using tissue paper at an interval of 2, 4 and 8 h. Swelling index of optimized formulations was determined from formula^51^

9 $$ \% {\rm{Swelling}}\,{\rm{Index}}=[({{\rm{w}}}_{{\rm{t}}}-{\rm{w}})/{\rm{w}}]\times 100$$

where w_t_ is sample weight after time t and w is initial sample weight

Average particle size and surface charge of samples suspended in HCl buffer pH 1.2 was evaluated after 2 h by dynamic light scattering technique (Malvern Metasizer, UK and Zetasizer Nano ZS, Malvern instruments, UK). Each reported value was average of three independent samples.

### Dissolution testing

The drug release from optimized DRC was determined using bio-relevant and compendia dissolution test method. The amount of drug released in mouth before swallowing of DRCs can be determined by assessing drug release under bio-relevant conditions of mouth^52^. DRC equivalent to 50 mg TAP placed in dialysis membrane bag was exposed for 3 min with 5 ml of simulated salivary fluid pH 7.4 (12 mM potassium dihydrogen phosphate, 40 mM sodium chloride and 1.5 mM calcium chloride in 1000 ml of distilled water adjusted to pH 7.4) to mimic bio-relevant conditions comparable to mouth^52^. However, dissolution fluid volume was small enough for sampling during study. Therefore, test sample amount and dissolution fluid volume was increased to same extent i.e. DRC equivalent to 500 mg TAP in 50 ml simulated salivary fluid pH 7.4 maintained at 37 ± 2 °C. Aliquots (2 ml) were withdrawn followed by replacement with fresh buffer at an interval of 0.5, 1.0, 1.5, 2.0, 2.5 and 3 min respectively and analysed spectrophotometrically at 271 nm.

Drug release from DRC in stomach was evaluated using compendia dissolution method since DRC remain in mouth for a very short duration. DRC equivalent to 50 mg of drug placed in dialysis membrane bag was immersed in HCl buffer pH 1.2 (900 ml) (250 ml of 0.2 M potassium chloride and 425 ml of 0.2 M HCl mixed and diluted with distilled water to 1000 ml) used as dissolution media maintained at 37 ± 2 °C with uniform stirring (50 rpm) in USP type II dissolution apparatus. Aliquots (2 ml) were withdrawn at regular intervals followed by replacement with fresh buffer up to 10 h and analysed spectrophotometrically at 271 nm.

### Stability study of optimized DRCs

Optimized DRCs were stored in an amber coloured glass container fitted with polypropylene cap. Packed containers were stored in stability chambers (FROILABO-SP 540BVEHF, France) under accelerated (40 ± 2 °C/75% ± 5% RH) and real time (30 ± 2 °C/65% ± 5% RH) stability testing conditions for 3 months and accessed for stability according to ICH guidelines^53^. After an interval of 1, 1.5 and 3 months’ storage, samples were analysed for *in vitro* release behaviour under bio-relevant conditions of mouth (simulated salivary fluid pH 7.4) and stomach (HCl buffer pH 1.2) respectively along with their visual appearance.

### *In vivo* study

The experimental protocol for conducting *in vivo* taste evaluation, pharmacokinetic and behavioural pharmacodynamics study was approved by Institutional Animal Ethical Committee of Banasthali Vidyapith, Rajasthan, India (574/GO/ReBi/S/02/CPCSEA). All animal experiments were conducted according to guidelines of Council for the purpose of control and supervision of experiments on animals (CPCSEA), Ministry of Social Justice and Empowerment, Government of India. Wistar rats (180 ± 20 g) were inhabited at temperature of 22 ± 2 °C, 55 ± 5% RH on a 12 h dark/light cycle with free access of food (standard synthetic pellet diet) and water *ad libitum*.

### Taste evaluation

Taste evaluation of TAP and optimized TAP-KyronT-134 complex was determined according to rat behavioural avoidance taste model. The model was based on the assumption that bitterness of solution governs the drinking frequency of water-deprived rats^54^. Animals were deprived of free access to food and water 24 h prior to experiment. Animals were randomly distributed into six groups (n = 6 rats per group). Each group-received sample orally i.e. group I: distilled water; group II: drug-KyronT-134 complex dispersion; group III: 10 µg/ml drug solution; group IV: 20 µg/ml drug solution; group V: 30 µg/ml drug solution and group VI: 40 µg/ml drug solution. According to study protocol, wistar rats of respective group were permitted to lick the samples viz. water, optimized drug-KyronT-134 complex dispersion and different concentration of drug solution (10–40 µg/ml) for 8 seconds respectively. DRC equivalent to 20 mg of drug was dispersed in distilled water (10 ml) just 5 min prior providing access of samples to wistar rats during *in vivo* taste evaluation study. The numbers of licks were determined by lickometer having electrophysiological setup. The percentage reduction in licking of test sample compared to water was determined according to formula^55^

10 $${\rm{Reduction}}\,{\rm{of}}\,{\rm{licking}}\,( \% )={[(N}_{{\rm{W}}}-\text{NT})/{{\rm{N}}}_{{\rm{W}}}]\times 100$$

where N_W_ is number of licks to water and N_T_ is number of licks to test sample

### Pharmacokinetic evaluation

Wistar rats were fasted overnight with free access to water *ad libitum* before commencement of study. Fasted animals were segregated into two groups (n = 6). Group I received TAP solution (3 mg/Kg) while group II received optimized DRC (dose equivalent to 3 m/Kg TAP). Both groups received respective treatment orally once. Blood samples (250 µl) were collected into heparinized tubes from retro-orbital plexus at an interval of 0.25, 0.5, 0.75, 1, 2, 4, 6 and 8 h. After each blood sampling, equal amount of normal sterile saline solution was injected to equilibrize blood loss volume. Plasma was isolated by centrifuging blood samples at 5000 rpm for 10 min at 4 °C. Thereafter, plasma samples were analysed for drug content by RP-HPLC. The HPLC system (LC-2010CHT; Shimadzu, Japan) equipped with UV-Vis detector (Shimadzu Co., Kyoto, Japan) and C_18_ column (Phenonmex column, 250 × 4.6 mm, 5 µm) was utilized for analysing drug concentration in plasma samples. Mobile phase consisting of a mixture of 0.1% formic acid and acetonitrile (75:25 v/v) was pumped at a flow rate of 1 ml/min at 25 ± 2 °C to elute the samples. Mobile phase was filtered through 0.45 µm Millipore membrane filter and de-aerated before use. Samples eluted were analysed at 271 nm^56^.

The observed pharmacokinetic data was analysed by non-compartmental pharmacokinetic analysis using WinNonlin (5.1: Pharsight, Mountain View, CA).

## Pharmacodynamics evaluation

### Development of peripheral neuropathic pain model

Peripheral neuropathic pain in wistar rats was induced by chronic constriction injury model^57^. Briefly, hairs from thigh region of left hind paw shaved with subsequent topical skin sterilization with Betadine^TM^. Consecutively, lateral surface of thigh skin incised to expose left sciatic nerve after grooving biceps femoris muscle under isoflurane anaesthesia (3% induction and 1.5% maintenance). Sciatic nerve ligated proximal to its trifurcation carefully without disrupting epineural circulation with four ligatures (silk black 4–0) at a distance of 1.5 mm under dissecting microscope (Olympus, Japan). Homeostasis was completed. Muscle and skin layers then sutured separately with subsequent application of topical antibiotic. Sham control group animals were subjected to surgical procedure during which sciatic nerve was exposed but neither ligated nor lesioned. Animals were euthanized after behavioural testing.

### Behavioural mechanical anti-allodynia activity

Nociceptive threshold to mechanical allodynia was determined using electronic von Frey pressure algometer (Model 2390; IITC Life Science Inc., Woodland Hills, CA, U.S.A.) on 7, 14 and 21^st^ day. Animals were divided into four groups each comprising of 6 wistar rats (n = 6). Group I: sham control treated with 0.5% w/v carboxymethyl cellulose *p.o*.; Group II: chronic constriction injury control treated with 0.5% w/v carboxymethyl cellulose *p.o*.; Group III: TAP solution (3 mg/Kg) treated chronic constriction injury; and Group IV: DRC dispersion (equivalent to 3 mg/Kg TAP) treated chronic constriction injury during the period of study. Animals were acclimatized in plexiglas box with wire mesh bottom for 15 min. Hand held force transducer von Frey probe with 0.7 mm^2^ circular tip was manually applied to induce mechanical allodynia on mid plantar area of hind paw. Force was increased constantly at a rate of 0.05 N/sec until animals showed escape response. Stimulus intensity was recorded automatically on paw withdrawal with electronic von Frey pressure algometer. The mechanical withdrawal threshold was explained as minimum force needed for eliciting reproducible paw withdrawal reflux^58^. Mechanical withdrawal threshold was average of three repeated measurements within 10 min interval. Paw withdrawal threshold was determined after oral administration of TAP solution (3 mg/Kg) or DRC (equivalent to 3 mg/Kg TAP) on each test day to a period of 30, 60, 120, 180 and 240 min.

### Statistical analysis

All results are presented as mean ± SD. Statistical analysis of data was performed using ANOVA followed by Holm-Sidak test through Sigma plot 12.0 software (Systat Software Inc CA, USA) to ascertain significant difference at p value less than 0.05. All the experiments were performed in triplicate. All data generated and analysed during this study are included in the article and in supplementary information file.

## Conclusion

Taste masking of the TAP HCl was successfully achieved by preparing DRC using ion exchange resin in batch process. *In vitro* release study and rat behavioural avoidance test model confirmed taste masking of drug in DRC. Among the ion exchange resins used in study, Kyron T-134 showed maximum drug loading and formed stable DRC facilitating prolonged release of drug up to 10 h. Sustained drug release characteristics of optimized formulation prolonged *in vivo* mean residence time of TAP facilitating improved bioavailability and neuropathic mechanical anti-allodynia activity. Conclusively, taste masked extended release DRCs can improve the palatability of extremely bitter drug along with probable potential of modifying the dosing regimen in allometric models of acute or chronic pain. The method used to prepare taste masked sustained release DRC is simple and economical as does not require special equipment. Therefore, this technique can be useful for preparing taste masked extended release DRCs of variety of drugs that can improve quality of patients.

## Electronic supplementary material

Supplementary information
