# Supplementary material for: Improved therapeutic potential of tapentadol employing cationic exchange resins as carriers in neuropathic pain: evidence from pharmacokinetic and pharmacodynamics study
Source: Sci Rep. 2018 Feb 12;8:2812. doi: 10.1038/s41598-018-21214-2 (PMC5809392; doi:10.1038/s41598-018-21214-2)
Supplement: Supplementary file 1 — Supplementary information [file 41598_2018_21214_MOESM1_ESM.pdf]

Manuscript Title: Improved therapeutic potential of tapentadol employing cationic exchange resins as carriers in neuropathic pain: evidence from pharmacokinetic and pharmacodynamics study

Authors : Manu Sharma,[*] Ranju Soni

Department of Pharmacy, Banasthali Vidyapith, Banasthali, Rajasthan, India-304022

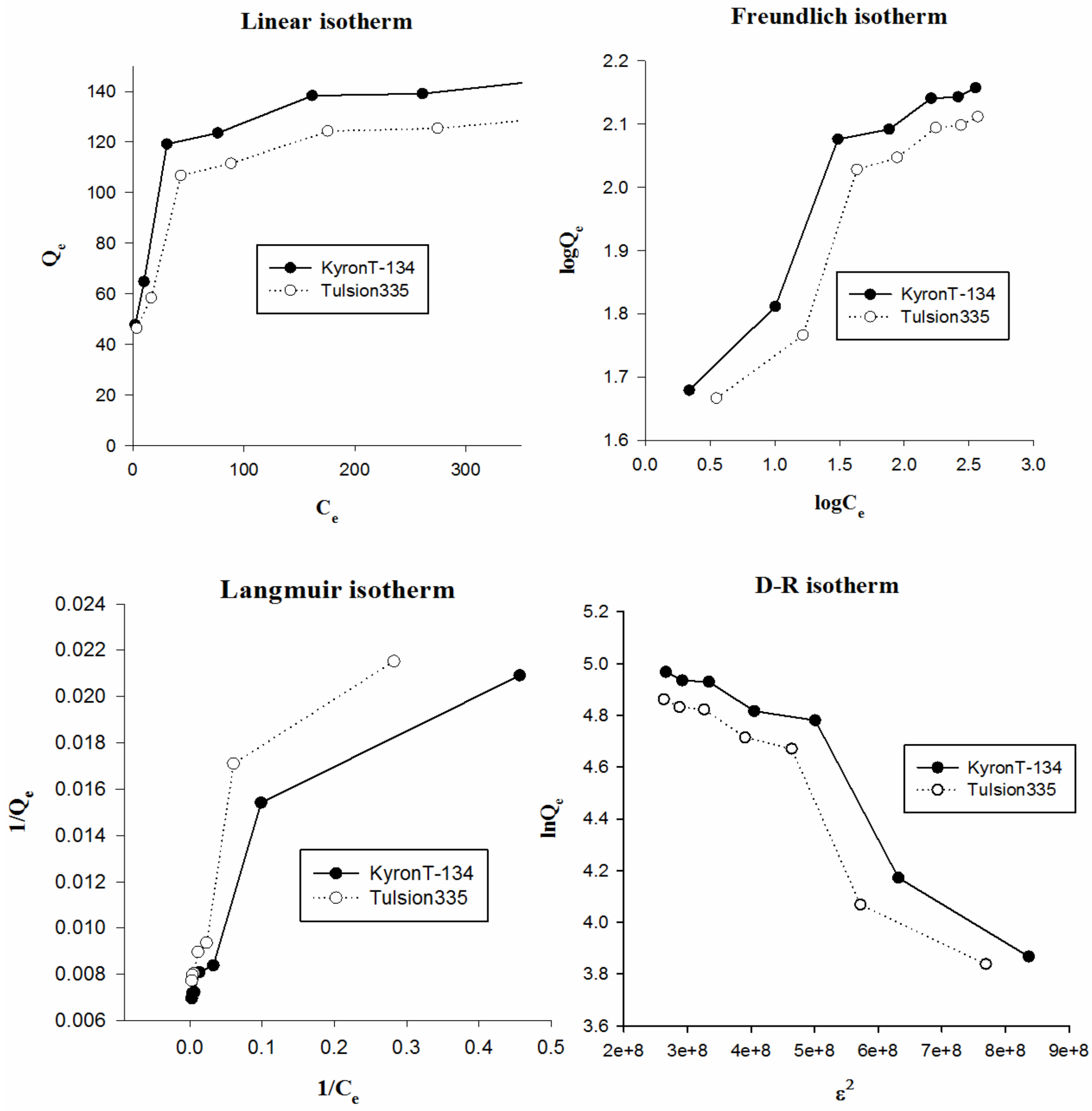

Supplementary Figure S1: Adsorption isotherms of TAP on cationic exchange resins viz. KyronT-134 and Tulsion335 respectively.

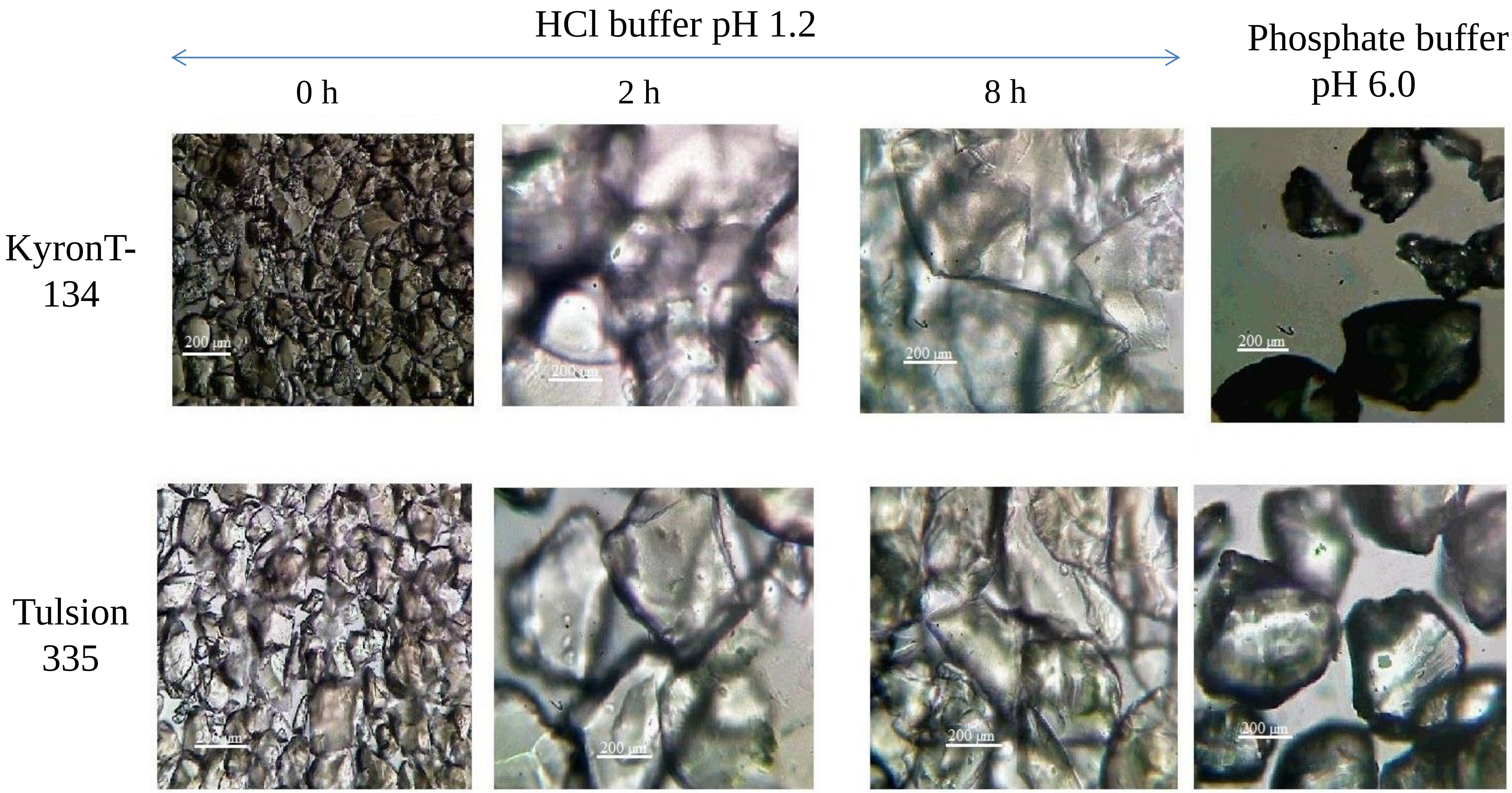

Supplementary Figure S2: Impact of time and pH on swelling and aggregation of optimized DRCs of KyronT-134 and Tulsion335 respectively.
